# SeqAn An efficient, generic C++ library for sequence analysis

**DOI:** 10.1186/1471-2105-9-11

**Published:** 2008-01-09

**Authors:** Andreas Döring, David Weese, Tobias Rausch, Knut Reinert

**Affiliations:** 1 Algorithmische Bioinformatik, Institut für Informatik, Takustr. 9, 14195 Berlin, Germany; 2 International Max Planck Research School for Computational Biology and Scientific Computing, Ihnestr. 63 – 73, 14195 Berlin, Germany

## Abstract

**Background:**

The use of novel algorithmic techniques is pivotal to many important problems in life science. For example the sequencing of the human genome [1] would not have been possible without advanced assembly algorithms. However, owing to the high speed of technological progress and the urgent need for bioinformatics tools, there is a widening gap between state-of-the-art algorithmic techniques and the actual algorithmic components of tools that are in widespread use.

**Results:**

To remedy this trend we propose the use of SeqAn, a library of efficient data types and algorithms for sequence analysis in computational biology. SeqAn comprises implementations of existing, practical state-of-the-art algorithmic components to provide a sound basis for algorithm testing and development. In this paper we describe the design and content of SeqAn and demonstrate its use by giving two examples. In the first example we show an application of SeqAn as an experimental platform by comparing different exact string matching algorithms. The second example is a simple version of the well-known MUMmer tool rewritten in SeqAn. Results indicate that our implementation is very efficient and versatile to use.

**Conclusion:**

We anticipate that SeqAn greatly simplifies the rapid development of new bioinformatics tools by providing a collection of readily usable, well-designed algorithmic components which are fundamental for the field of sequence analysis. This leverages not only the implementation of new algorithms, but also enables a sound analysis and comparison of existing algorithms.

## Background

**Figure 1:**
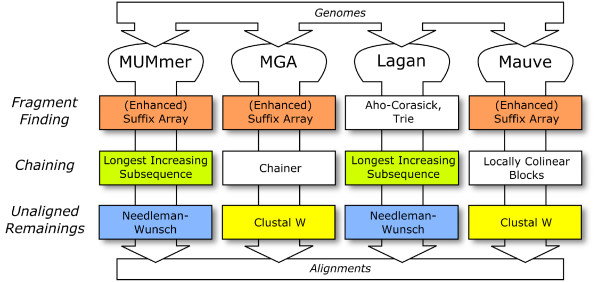
Genome comparison tools and their algorithmic components.

Biological sequence analysis is the heart of computational biology. Many successful algorithms (e.g., Myers' bit-vector search algorithm [2], BLAST [3]) and data structures (e.g., suffix arrays [4], *q*-gram based string indices, sequence profiles) have been developed over the last twenty years. The assemblies of large eucaryotic genomes like *Drosophila melanogaster *[5], human [1], and mouse [6] are prime examples where algorithm research was successfully applied to a biological problem. However, with entire genomes at hand, large scale analysis algorithms that require considerable computing resources are becoming increasingly important (e.g., Lagan [7], MUMmer [8], MGA [9], Mauve [10]). Although these tools use slightly different algorithms, nearly all of them require some basic algorithmic components, like suffix arrays, string searches, alignments, or the chaining of fragments. This is illustrated in Fig. 1 for the case of genome comparison tools. However, it is non-trivial to obtain efficient implementations of these components. Therefore, suboptimal data types and *ad-hoc *algorithms are frequently employed in practice, or one has to resort to stringing standalone tools together. Both approaches may be suitable at times, but it would clearly be much more desirable to use an integrated library of state-of-the-art components that can be combined in various ways, either to develop new applications or to compare alternative implementations. In this article we propose SeqAn, a novel C++ library of efficient data types and algorithms for sequence analysis in computational biology.

In other fields, software libraries have greatly advanced the transfer of algorithmic knowledge to the tool programming process. Two of the best known examples are the LEDA library [11] for algorithms on graphs and efficient data types and the CGAL library [12,13] for computational geometry. In bioinformatics, a comparable library is still missing although there is a need for integrated implementations of algorithms for aligning sequences, computing substring indices in primary and secondary memory, or filter algorithms. In addition, a library that adheres to the principles of algorithm engineering is essential as a means to test and compare existing tools as well as to evaluate the results from algorithmic research. The lack of such a library becomes evident when reviewing the related work of the past years.

A few C++ libraries with sequence analysis features have already been developed, including Bio++ [14], Libcov [15], the Bioinformatics Template Library (BTL) [16], the NCBI C++ Toolkit [17], or the Sequence Class Library (SCL) [18]. Bio++ is the most comprehensive library providing re-usable components for phylogenetics, molecular evolution, and population genetics. The sequence analysis part is, however, limited to basic import/export capabilities and string manipulations. In contrast to SeqAn, which is based upon the generic programming paradigm, Bio++ is a purely object-oriented library, favoring ease of development over performance and scalability. Libcov focusses on phylogenetics and clustering algorithms. It offers only basic data structures to handle sets of sequences. Alignment algorithms, database indices, or scoring matrices are not provided. The BTL emphasizes basic *mathematical *algorithms and data structures. It currently comprises graph classes and linear algebra algorithms but only a single sequence alignment algorithm, Needleman-Wunsch [19] with cubic running time. The NCBI C++ Toolkit also offers, beside other things, some sequence analysis functionality, e.g. alignment algorithms. The SCL, providing some basic sequence analysis components, is to our knowledge not activly developed anymore.

Besides these C++ libraries, we are aware of alternative approaches like BioPerl [20] or BioJava [21]. The main purpose of BioPerl is to ease the development of sequence analysis pipelines by providing an interface to already existing tools. BioJava on the other hand is suited for developing new sequence analysis tools by providing many relevant data structures and algorithms and as such is from the overall goals perhaps closest to SeqAn. Some algorithms are shared by both libraries (like Needleman-Wunsch and Smith Waterman). A closer inspection, however, reveals that BioJava does only offer a small part of SeqAn's functionality (no indices, no algorithms for de novo motif search, no algorithms for multiple alignment, etc.). Hence both libraries are in this sense complementary. In addition, we show in the result section that our implementations are for standard alignment problems by a factor of 6 to 350 times faster and by a factor of 600 to 1500 times more space efficient.

The exposition is structured as follows: To emphasize the usefulness of SeqAn, this article centers around the contents of the library, described in Section and the practical application of SeqAn, exemplified in Section. In the following section we start by giving a brief outline of the design principles SeqAn is based on.

## Implementation

### Library Design

For developing the basic design, SeqAn has gone through an extensive conceptual phase in which we evaluated many designs and prototypic implementations. SeqAn has now a generic programming design that guarantees high performance, generality, extensibility, simplicity, and easy integration with other libraries. This design is based on four design principles which we will describe now.

#### Generic Programming

SeqAn adopts the generic programming paradigm that proved to be a key technique for achieving high performance algorithms in the C++ standard library [22]. Generic programming refers to a special style of programming where concrete types are substituted by exchangeable template types. Hence, classes and algorithms are written only once, but can be applied to different data types.

#### Global Function Interfaces

SeqAn uses global functions instead of member functions to access objects (we act here on an advice of [23], see Section 6.10.2.). This strategy improves the flexibility and the scalability of our library, since global functions, unlike member functions, can be added to a program at any time and without changing the existing code. Moreover, global function interfaces enable us to incorporate the C++ built-in types and handle them like user defined types. It is even possible to adapt arbitrary interfaces, i.e. of classes that are implemented in external libraries, to a common interface by using small global functions called *'shims' *(Chapter 20 in [24]). Algorithms that access objects only via global functions can therefore be applied to a great variety of types, including built-in types and external classes.

#### Traits

Generic algorithms usually have to know certain types that correspond to their arguments: An algorithm on strings may need to know which type of characters are stored in the string, or what kind of iterator can be used to browse it. SeqAn uses type traits [25] for that purpose. In C++, trait classes are implemented as class templates that map types or constants given by template arguments to other types but also other C++ enties like constants, functions, or objects at compile time. Most of the advantages we already stated for global functions also apply to traits, i.e. new traits and new specializations of already existing traits can be added without changing other parts of the library.

#### Template Argument Subclassing

SeqAn uses a special kind of hierarchical structure that we call 'template argument subclassing', which means that different specializations of a given class template are specified by template arguments. For example, String<Compressed> is a subclass of String in the sense that all functions and traits which are applicable to String can also be applied to String<Compressed>, while it is possible to overload some functions especially for String<Compressed>. The rules of C++ overload resolution guarantee that the compiler always applies the most specific variant out of all existing implementations when an algorithm or trait has been called. This approach resembles class derivation in standard object-oriented programming, but it is often faster, because it does not require a type conversion for a subclass calling a function that is already defined for the base class, and since the actual type of the object used in a function is therefore already known at compile time, it is not necessary to detect it at run time using virtual functions. Non-virtual functions have the advantage that C++ compilers can use function inlining to save their overhead completely. Template argument subclassing enables us both to specialize functions and to delegate tasks soundly to base classes while still maintaining static binding.

#### Design Goals

These design principles support our design goals in the following way:

• **Performance: **The library produces code that is competitive with manually optimized programs. Template argument subclassing makes it possible to plug in optimized specializations for algorithms whenever needed. Our generic programming design also speeds up the code in avoiding unnecessary virtual function calls.

• **Generality: **All parts of the library are as flexible as possible. Algorithms can be applied to various data types and new types can be added if necessary. For example, generic alignment algorithms in SeqAn work on strings for arbitrary alphabets. However, specialized implementations that make use of certain attributes of the alphabet can still be developed using template argument subclassing.

• **Integration: **SeqAn components are designed to fulfill the requirements specified in the C++ standard. In addition, SeqAn easily interacts with other libraries because the global interface can be expanded. Hence, algorithms and classes of other libraries are at hand.

• **Extensibility: **The open-closed principle ('Be open for extension but closed for modifications!') is satisfied in so far as it is possible to extend the library by simply adding new code. SeqAn has this feature because it relies on stand-alone global functions and traits that can be added at any time without changing the existing code.

• **Simplicity: **While a pure object-oriented library may be more familiar to some users, SeqAn is still simple enough to be used even by developers with average skills in C++.

### Library Contents

**Figure 2:**
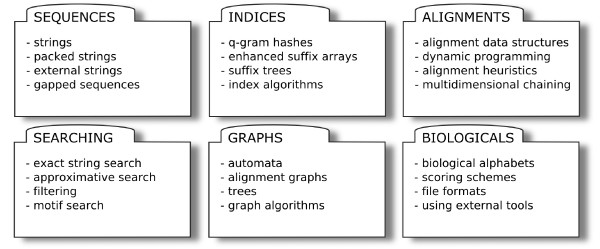
SeqAn Contents Overview.

SeqAn is a software library that is supposed to cover all areas of sequence analysis. Fig. 2 gives an overview of the contents of the library in the current state.

#### Sequences

The storage and manipulation of sequences is essential for all algorithms in the field of sequence analysis. In SeqAn, sequences are represented as strings of characters over various alphabets. Multiple string classes for different settings are available: Large sequences can be stored in secondary memory using external strings, bit-packed strings can be used to take advantage of small alphabets, or strings allocated on the stack can be used to guarantee fast access. String modifiers can be used to implement distinct views on a given sequence without copying it. A string segment, for instance, is a string modifier used to get access to an infix, suffix, or prefix of a given sequence.

#### Alignments

Alignments require the insertion of gaps into sequences. SeqAn does not actually insert these gaps directly into the sequence but treats them separately. The benefit is twofold: A single sequence can be used in multiple alignments simultaneously and the actual alphabet of the string must not include a special gap character. SeqAn offers both pairwise and multiple sequence alignment algorithms. Algorithms can be configured for different scoring schemes and different treatments of sequence ends (e.g, ends free-space alignments). In the pairwise case, local and global alignment algorithm are available. Besides the classical Needleman-Wunsch algorithm [19], more sophisticated algorithms are available, including an affine gap cost alignment [26] and Myer's bit vector algorithm [2]. Moreover, SeqAn offers efficient algorithms to chain alignment fragments [27,28]. We are also currently integrating code for motif finding in multiple sequences.

#### Indices

The *enhanced suffix array (ESA) *[29] is probably the most fundamental indexing data structure in bioinformatics with various applications, e.g., finding maximal repeats, super maximal repeats, or maximal unique matches in sequences. An enhanced suffix array is a normal suffix array extended with an additional *lcp *table that stores the length of the longest common prefix of adjacent suffixes in the suffix array. SeqAn offers an ESA that can be build up in primary or in secondary memory, depending on the sequence size. The user has two choices to access the ESA, either as a regular suffix array or as a suffix tree. The later view on an ESA is realized using the concept of iterators that simulate a tree traversal. A more space and time efficient data structure for top-down traversals through only parts of the suffix tree is the lazy suffix tree [30] which is also implemented in SeqAn. Besides the sophisticated ESA, simpler indices are available, including basic hash tables like gapped- and ungapped q-gram indices (for their use see [31-33]).

#### Searching

Flexible pattern matching algorithms are fundamental to sequence analysis. Exact and approximate string matching algorithms are provided. For the exact string matching task, SeqAn offers the algorithms Shift-And, Shift-Or, Horspool, Backward Oracle Matching, and Backward Nondeterministic Dawg Machine [34]. For searching multiple patterns, SeqAn currently supports the Multiple Shift-And, the Set Horspool, and the Aho-Corasick algorithm [34]. Myer's bit vector algorithm [2] can be used for approximate string matching. Note that SeqAn's index data structures can naturally be used to search for strings as well.

#### Graphs

Graphs are increasingly important to a number of bioinformatics problems. Prime examples are string matching algorithms (e.g., Aho-Corasick, Backward Oracle Matching [34]), phylogenetic algorithms (e.g., upgma, neighbor joining tree [35]), or alignment representations [36]. Hence, we decided to include our own graph type implementation, including directed graphs, undirected graphs, trees, automata, alignment graphs, tries, wordgraphs, and oracles. Graph algorithms currently comprise breath-first search, depth-first search, topological sort, strongly-connected components, minimum spanning trees (e.g., Prim's algorithm, Kruskal's algorithm), shortest path algorithms (e.g., Bellman-Ford, Dijkstra, Floyd-Warshall), transitive closure, and the Ford-Fulkerson maximum flow algorithm [37]. Trees are heavily used in clustering algorithms and as guide trees during a progressive multiple sequence alignment. Alignment graphs are used to implement a heuristic multiple sequence alignment algorithm, which is similar to T-Coffee [38] but makes use of segments and a sophisticated refinement algorithm [39] to enable large-scale sequence alignments.

#### Biologicals

Besides the fundamental alphabets for biological purposes, like DNA or amino acids, SeqAn offers different scoring schemes for evaluating the distance of two characters, e.g., PAM, and BLOSUM. SeqAn also supports several file formats that are common in the field of bioinformatics, e.g., FASTA, EMBL, and genbank. Is is possible the access (e.g. to search) sequence data stored in such file formats without loading the whole data into memory. The integration of external tools (e.g., BLAST) and the parsing of metainformation is ongoing work.

## Results

We anticipate two different user groups for SeqAn. The first group is the bioinformatics practitioner with some programming knowledge who wants to quickly prototype efficient tools for analyzing genomic or protein sequences *using *SeqAn. The other prototypic user is the algorithmicist who is proficient in programming *in *SeqAn and wants to test and compare an algorithmic component for a specific well-defined algorithmic problem.

The next examples will demonstrate how things could be done in SeqAn. We would like to point out the very good performance of SeqAn as well as the fact that the necessary code is small, easy to understand, generic, and greatly profits from using an integrated algorithmic library.

### Example: String Matching

**Figure 3:**
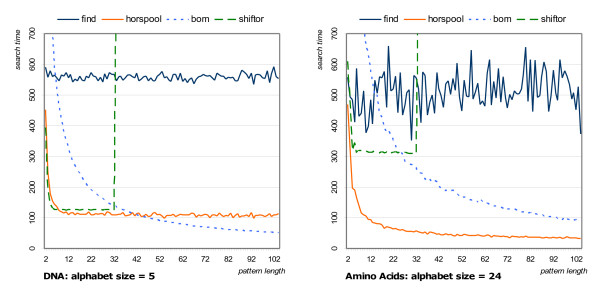
**Runtimes of String Matching Algorithms**. We compared three exact string matching algorithms from SeqAn with the member function basic_string::find of the standard library, as it was implemented for Microsoft Visual C++. The left figure shows the runtimes (in *ms*) for searching a DNA sequence (human chromosome 21), the right figure for searching a proteine database. The search pattern was taken randomly from the sequence. The figures show the average time needed to find all occurrences of patterns of a given length.

We start with a small example of how SeqAn could be used as an experimental platform to test various implementations to solve the same algorithmic problem. In Fig. 3 we show the results of a runtime comparison between three string matching algorithms implemented in SeqAn and the find method for strings from the standard template library. Different pattern lengths and alphabet sizes were used. It turned out that there is always string matching routines in SeqAn that is faster than standard library code. This demonstrates that the coding standard used in SeqAn is competitive to a widely used STL implementation. Note that none of the tested algorithms performs the best for all settings. A library like SeqAn makes it possible to switch between different algorithms easily, so users can apply the best algorithm depending on the requirements. Moreover, SeqAn can act as an experimental platform to compare new string matching methods with the set of well known algorithms present in SeqAn.

### Example: Global Alignments

Now we switch to a more biologically motivated example: Computing a global alignment between two given DNA sequences with minimal edit distance. Alignment problems are very popular in the biological context. Even libraries with little sequence analysis content support one or more relevant functions that are mostly based on the dynamic programming approach by Needleman and Wunsch [19], which is certainly one of the most popular algorithms in bioinformatics. Gotoh's algorithm [26] extends it by affine gap cost models. Some libraries also support Hirschberg's algorithm [40], another well-known alignment method that takes only linear space.

Table 1 lists time and space requirements for aligning the genomes of two human influenca viruses. The results show that SeqAn can compete with other libraries regardeless of scoring scheme and gap cost model. In the case of edit distance, SeqAn actually beats all competitors with a special algorithm that combines Hirschberg's algorithm with Myers' bitvector algorithm [2] to compute an optimal alignment one order of magnitude faster than all other programs we observed.

**Table 1:** Runtimes and internal space requirements for computing sequence alignments. The table shows average time and space requirements for aligning the genomes of two human influenca viruses, each of length about 15.6 kbp. Runtimes printed in bold face show for each library the time of the fastest algorithm for computing an alignment using edit distance.

	**linear gap costs**		**affine gap costs**	
	time (s)	space (MB)	time (s)	space (MB)
**SeqAn**				
Needleman-Wunsch	3.3	236	6.3	236
Hirschberg			14.7	4
Myers-Hirschberg	**0.2**	3		
**NCBI C++ toolkit**				
Needleman-Wunsch			**4.0**	245
Hirschberg			6.6	14
**Bio++**	**13.4**	2100	28.0	≈6000
**BTL**			**96162**	933
**BioJava**	**76**	2000	93	≈6000

### Example: MUMmer

In this example, we want to convince the reader that programming *using *SeqAn is not difficult and that SeqAn is simple enough to meet the needs of the first user group while it is efficient and generic enough to allow expert users to use SeqAn as an experimental platform for testing algorithmic components.

We choose as an example the well-known MUMmer [8] tool and show in the listing (see Additional File 1) an implementation of a (simplified) version of the tool which reads a number of sequences and computes all maximal unique matches (MUMs) of length at least 20. For the sake of exposition we only show code pieces that are illustrative for SeqAn.

A MUM of a set of sequences is a subsequence that occurs exactly once in each sequence and that is not part of any longer such subsequence. To find MUMs MUMmer builds a suffix tree [41] of a reference sequence and streams one or more query sequences against it. The downside of designating one sequence as the reference is that matches are only unique in the reference sequence but not necessarily in the query sequence. To guarantee the uniqueness of a match in all sequences it is necessary to construct a generalized suffix tree. A generalized suffx tree is a suffix tree of the concatenated sequences seperated by unique characters [42]. It is the primary index data structure in SeqAn and based on an enhanced suffix array [43]. In the first part of the example (Additional File 1: Listing 1) we build a string index for a set of strings consisting of characters from the Dna5 alphabet, which is an extension of the Dna alphabet including the 'N' character. SeqAn supports a number of different alphabets of biological relevance (e.g., Dna, Amino Acid, or compressed amino acid alphabets). All these alphabets enable us to store sequences efficiently because of the reduced number of characters compared to normal text. The index is first resized to the appropriate number of sequences. Subsequently, the sequences are imported using the Fasta read function and simultaneously, these sequences are added to the index, which is our enhanced suffix array.

SeqAn provides iterators that make it possible to traverse the enhanced suffix array like a suffix tree in a bottom-up fashion. This is illustrated in Additional File 1: Listing 2. The iterator it visits each node *u *in the generalized suffix tree. To find a MUM *u*, it suffices to test whether *u *occurs exactly 2 times (line 16), at most once in each sequence (line 18), and cannot be extended to the left (line 19) (see Chapt. 3.4, [43]). If the length of the representative string of *u *is also at least 20 (line 17) we report the position and length of the MUM *u *(lines 26–31). Assuming a computational biologist is not all too interested in these algorithmic details but in performance and a simple interface, SeqAn provides specialized iterators to get all MUMs (Additional File 1: Listing 3), maximal or supermaximal repeats, or MultiMEMs [43]). Since performance is a crucial issue in any kind of sequence analysis task, we compared our code example with the latest MUMmer release [8] and Multimat of the MGA distribution [9]. To the best of our knowledge these are the only tools to find MUMs. None of the libraries introduced in chapter support generalized suffix trees or even algorithms on suffix trees, like those to find MUMs. Our testset consisted of various bacteria strains and vertebrate chromosomes.

Table 2 readily reveals that MUMmer is about twice as fast as SeqAn on the 2 sequence datasets and that it uses only half of the space. This is, however, not suprising because MUMmer's index represents only one sequence whereas the generalized suffix tree implemented in SeqAn builds an index over all sequences. But in contrast to SeqAn and MGA, MUMmer is not able to find real MUMs between more than 2 sequences. Similar to SeqAn, MGA also constructs a generalized suffix tree of all sequences and its memory consumption is approximately equal to SeqAn's. However, SeqAn outperforms MGA on all datasets and surprisingly, MGA even takes more than 1 hour on the E. coli strains.

**Table 2:** Runtimes and internal space requirements for finding MUMs. We compared MUMmer 3.19 [8], MGA [9], and SeqAn for different DNA sequences on a 3.2 GHz Intel Xeon computer with 3 GB of internal memory running Linux. Because MUMmer finds MUMs of not more than two sequences, its results on the Chlamydia and Escherichia coli strains are left empty. For the last dataset, we used SeqAn's external memory data strutures to limit the internal memory consumption.

**Species**		**MUMer**		**MGA**		**SeqAn**	
	size (Mbp)	time (m:s)	space (MB)	time (m:s)	space (MB)	time (m:s)	space (MB)
C. trachomatis D/UW-3/CX	1.043						
C. muridarum Nigg	1.073	-	-	0:06	33.8	0:04	31.6
C. trachomatis A/HAR-13	1.044						
							
E. coli K12	4.640						
E. coli O157:H7 str. Sakai	5.498						
E. coli CFT073	5.231	-	-	105:40	353.5	0:58	304.6
E. coli UTI89	5.066						
E. coli 536	4.939						
E. coli APEC O1	5.082						
							
H. sapiens (chr. 21)	46.94	2:25	568	4:44	1188	4:06	1307
M. musculus (chr. 16)	98.25						
							
H. sapiens (chr. 16)	98.25	2:55	1362	18:48	1500	5:38	1627
P. troglodytes (chr. 16)	88.83						
						**external string**	

H. sapiens (chr. 1)	247.2	insufficient memory		insufficient memory		66:20	510
M. musculus (chr. 1)	197.1						

Index data structures of whole genomes easily reach 10–30 GB, so they must rely on external memory. SeqAn provides such data structures. They can be used by simply exchanging the standard string of SeqAn by an external memory string in order to construct the generalized suffix tree in external memory. We did this for the last row of Table 1 and simply replaced Dna5String by String<Dna5, External<>> in the code example. This reduces the main memory space requirements of the algorithm at the expense of speed. However, this makes it possible to construct generalized suffix trees for sequences of nearly arbitrary length. As can be seen in Table 1, these external strings enable SeqAn to handle long sequences where MUMmer and MGA simply run out of memory.

## Conclusion

We presented a new software library with an efficient and generic design that addresses a wide range of problems in sequence analysis. SeqAn is under active development and we hope that it will become one of the standard platforms for algorithmic engineering at the interface of stringology, algorithm design and computational biology. Besides the planned extensions mentioned in Section, we are working on integrating external libraries and plan to intensify our collaborations with other research groups.

## Availability and requirements

SeqAn is freely distributed under the GNU Lesser General Public Licence (LGPL), both for academic and non-academic use. The library and its documentation can be downloaded from . All parts of the library are tested on Windows with Microsoft Visual Studio 2003 and 2005 and on Linux with G++ compilers version 3.0 and above.

### Competing interests

The author(s) declares that there are no competing interests.

## Authors' contributions

AD worked out the overall design of the library and implemented a large part of the kernel functionality. DW took responsibility mainly for the index data structures and the pipelining in SeqAn. The work of TR includes everything in the library that has to do with graphs. Programming SeqAn was team work, so all three programmers (AD, DW, and TR) left tracks in almost every part of the library. KR supervised the project. All authors participated equally in composing this manuscript.

## Supplementary Material

Additional file 1 **Listings**. The listings show C++ code that uses SeqAn to implement a simplified version of the well-known MUMmer tool [8]. Click here for file
