# Supplementary material for: SeqAn An efficient, generic C++ library for sequence analysis
Source: BMC Bioinformatics. 2008 Jan 9;9:11. doi: 10.1186/1471-2105-9-11 (PMC2246154; doi:10.1186/1471-2105-9-11)
Supplement: Additional file 1 — Listings. The listings show C++ code that uses SeqAn to implement a simplified version of the well-known MUMmer tool [8]. [file 1471-2105-9-11-S1.PDF]

Listing 1 - Building an index from two Fasta sequences

```
const char* filename[] = { "hs_ref_chr1.fa", "mm_ref_chr1.fa" };

Index< StringSet<Dna5String> >  index;
resize(indexText(index), 2);

for (int seq = 0; seq < 2; ++seq) {
    ifstream file(filename[seq], ios_base::in | ios_base::binary);
    read(file, indexText(index)[seq], Fasta());
    file.close();
}
```

Listing 2 - Finding MUMs using a bottom-up traversal of the suffix tree

```
typedef Index< StringSet<Dna5String> >  TIndex;
Iterator<TIndex, BottomUp<> >::Type     it(index);

while (true) {
    while (!atEnd(it) && !(
        (countOccurences(it) == 2) &&
        (repLength(it) >= 20) &&
        isUnique(it) &&
        isLeftMaximal(it)) )
    {
        goNext(it);
    }

    if (atEnd(it)) break;

    String< SAValue<TIndex>::Type > occs = getOccurences(it);
    orderOccurences(occs);

    cout << getValueI2(occs[0]) << "␣␣";    // MUM position in sequence 1
    cout << getValueI2(occs[1]) << "␣␣";    // MUM position in sequence 2
    cout << repLength(it) << endl;          // MUM length
}
```

Listing 3 - Specialized iterators to simplify the search for MUMs

```
typedef Index< StringSet<Dna5String> >  TIndex;
Iterator<TIndex, MUMs>::Type            it(index, 20);

for (; !atEnd(it); goNext(it)) {
    String< SAValue<TIndex>::Type > occs = getOccurences(it);
    orderOccurences(occs);

    cout << getValueI2(occs[0]) << "␣␣";    // MUM position in sequence 1
    cout << getValueI2(occs[1]) << "␣␣";    // MUM position in sequence 2
    cout << repLength(it) << endl;          // MUM length
}
```
